# A Differential Diagnosis of Haematuria following a Motor Vehicle Collision: Nutcracker Syndrome

**DOI:** 10.1155/2015/749182

**Published:** 2015-01-01

**Authors:** Gary Sharp, Derek Glenn

**Affiliations:** ^1^Trauma Department at St George Hospital, Kogarah, Sydney, NSW 2217, Australia; ^2^St George Hospital, Kogarah, Sydney, NSW 2217, Australia

## Abstract

A young female presented to the emergency department following a motor vehicle collision. She complained of left flank pain and was found to have haematuria. After investigation no trauma related injuries were identified. However, an incidental finding of nutcracker phenomenon/syndrome was made. Nutcracker phenomenon is a rare cause of haematuria resulting from nontraumatic compression of the left renal vein between the superior mesenteric artery and the aorta. It affects females more than males and its presentation can range from asymptomatic to debilitating haematuria, pelvic congestion in females, varicosities in males, and pain. No validated diagnostic criteria exist and treatment is usually surgical in those with debilitating symptoms or refractory anaemia.

## 1. Introduction

Nutcracker syndrome (NCS) was first described by Grant [1], who likened the impingement of the left renal vein (LRV) by the superior mesenteric artery (SMA) against the aorta to that of a nut within the jaws of a nutcracker. The first clinical case of NCS was acknowledged by El-Sadr and Mina in 1950 [2] and its management was first documented in 1974 [3].

A clear distinction between NCS, the* clinical manifestation* of mesoaortic compression of the LRV, and nutcracker phenomenon (NCP), the* anatomical* identification of mesoaortic compression of the LRV, exists [4–6]. Its rarity is represented through scant evidence with no validated diagnostic or therapeutic guidelines [5]. As such there is no data quantifying prevalence or incidence [4, 5]. NCP is subdivided into anterior and posterior subtypes. Anterior NCP refers to mesoaortic compression of the LRV whilst the rarer posterior NCP denotes compression of a retroaortic LRV between the aorta and vertebrae [4–6]. Only anterior NCP will be addressed hereafter.

**Figure 1:**
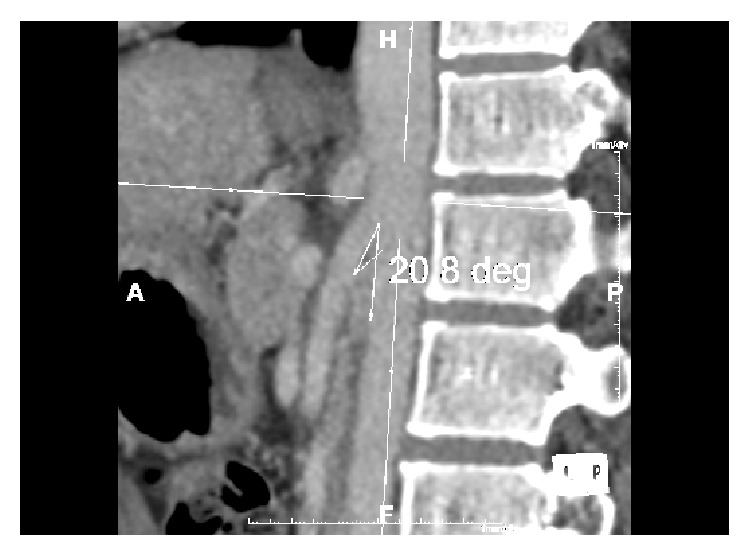
Sagittal contrast CT of our patient showing a mesoaortic angle of 20.8 degrees.

Females are most often affected in bimodal fashion [5, 7] with the first peak at 20–30 years and the second in middle age [5, 7]. It must be stressed however that both sexes and all ages can be affected [4, 5, 7]. Low body mass index is regarded as a risk factor of NCP [4, 5, 7] due to a paucity of retroperitoneal adipose tissue reducing the mesoaortic angle and/or causing posterior renal ptosis [5, 7]. Posterior renal ptosis refers to dorsal migration of the kidney and renal pelvis due to the aforementioned retroperitoneal adipose tissue paucity [4, 6, 7]. This posterior displacement stretches and compresses the LRV [6]. NCP may also arise due to anatomical variations such as a LRV that is more cephalad upon union with the inferior vena cava and as such is immediately inferior to the SMA or an SMA that instantly descends [5]. In healthy individuals the mesoaortic angle is reportedly between 38 and 90 degrees; in NCP it is suggested that this angle is greater than halved [5] (Figure 1). Other causes of LRV compression include pancreatic neoplasms, para-aortic lymphadenopathy, retroperitoneal tumours, aortic aneurysms, or fibrolymphatic tissue between the SMA and aorta [4, 5]. No genetic link has been identified [5–7].

## 2. Case Report

A 31-year-old female presented to the emergency department following a motor vehicle collision. On examination she complained of left flank tenderness. Her urine was positive for blood but beta human chorionic gonadotropin negative. Abdominal and pelvic contrast computed tomography imaging displayed no intra-abdominal sequelae of trauma. However, it demonstrated left renal vein compression between the superior mesenteric artery and aorta, known as nutcracker phenomenon, which can manifest as haematuria. Her haematuria unfortunately still persists and the best course of treatment is being evaluated. We have been unable to locate any literature regarding traumatic patients presenting with haematuria subsequently diagnosed as nutcracker phenomenon/syndrome. Here we review such a case.

## 3. Discussion

Haematuria is the most common presenting compliant of NCS and can manifest as macroscopic or microscopic dependent upon renal venous hypertension severity [4–7]. Compression of the LRV results in higher pressures distally which manifests as peri- and pararenal varicosities and collaterals [8] that communicate directly with the low pressure calyces [4–7, 9]. These thin walled collaterals often succumb and rupture with resultant haematuria [4–7, 9]. Pain is the second most common presenting complaint [5] and is reported as abdominal or flank in nature [5, 9]. Renal colic may also be present due to passage of clots along the left ureter [5, 7].

The LRV receives tributaries of the left adrenal, left gonadal, ureteral, and lumbar veins prior to joining the IVC. These veins usually have competent valves; when the pressure increases varicosities are formed [5] leading to gender specific complaints such as left sided varicocele in males [4–6, 9] and pelvic congestion in females [4–6, 9]. Pelvic congestion is more common in multiparous middle aged females [5]. Its prevalence is quoted as 5.5% in female NCS sufferers and pain is thought to arise due to inflammatory cascade initiation secondary to the hypertensive vasculature [4]. Pelvic congestion may present as dyspareunia, dysuria, or dysmenorrhoea [4–7] which rarely persist past menopause [7].

**Figure 2:**
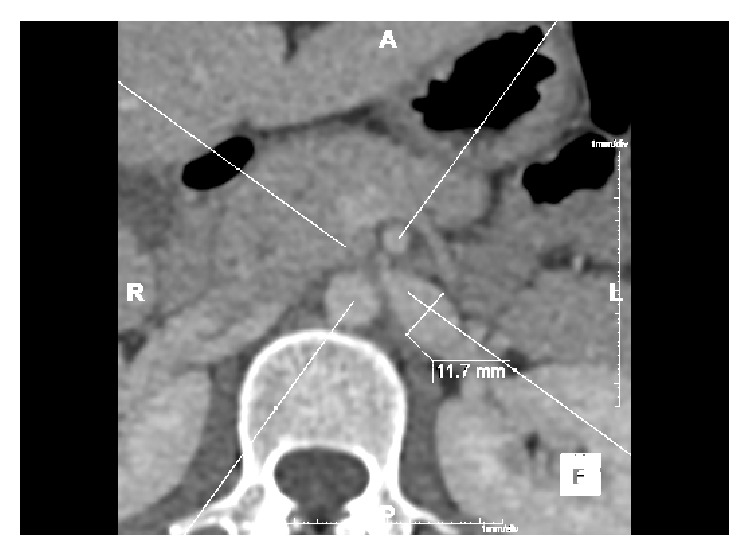
Axial contrast CT in our patient showing a grossly dilated LRV (11.7 mm) prior to impingement due to mesoaortic compression.

**Figure 3:**
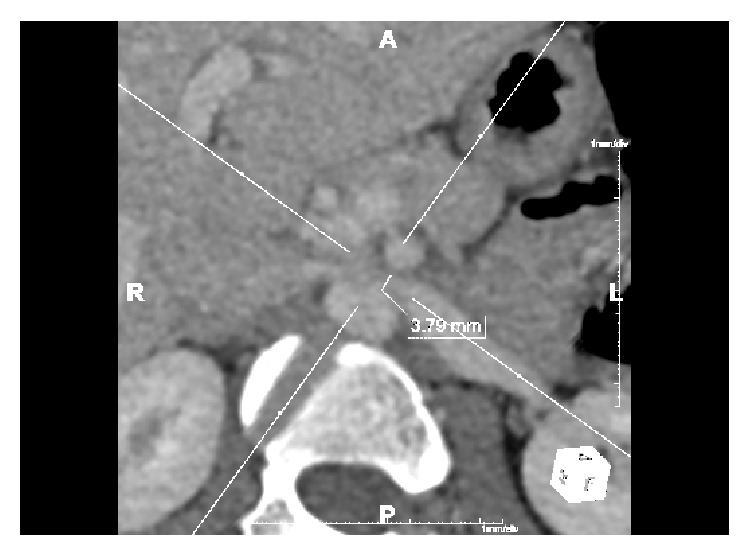
Axial contrast CT in our patient showing a LRV diameter of 3.79 mm due to mesoaortic compression. A reduction in diameter of ≥50%.

No validated diagnostic criteria exist [4, 7]. CT visualises LRV diameter [4, 7], collaterals, and the mesoaortic angle [9] but cannot measure velocity changes [4] that are characteristic of NCP. A LRV diameter of 4-5 mm is regarded as normal [4–6]. Authors suggest measuring LRV diameter prior to impingement and at the point of compression to evaluate the ratio, a reduction of ≥50% being diagnostic of NCP [6] (Figures 2 and 3).

Normally the pressure difference between the LRV and IVC is ≤1 mmHg [4, 5, 7]. LRV hypertension occurs when the gradient is ≥3.0 mmHg [5–7] culminating in renal venous collaterals and ultimately haemorrhage [6]. Many authors suggest venography to be “the definitive test for NCS” [7] or in fact the “gold standard” [4, 9], because it accurately measures renocaval pressure gradient [4]. Doppler ultrasound is good at measuring LRV/IVC pressure gradient [4] and coupled with its noninvasive nature some argue it should be the first investigation when NCS is suspected [5, 9].

No standardised treatment exists [4]. Conservative treatment is recommended for mild haematuria in those <18 years [4–7]. Surgical treatment focusses on decompressing renal hypertension [4] in those with refractory symptoms [4, 5, 7].

LRV transposition involves detaching the LRV from the inferior vena cava (IVC) and reattaching it caudally [9]. It is currently the most frequently utilised surgical treatment [9] and is regarded by many as the best procedure in terms of morbidity and outcome [7, 9]. This technique was, until recently, carried out via laparotomy but successful laparoscopic procedures have since been performed [4].

Other surrounding veins (gonadal, left adrenal, and left lumbar) can be isolated and ligated to enable adequate LRV mobilisation and tension-free anastomoses [9]. The original LRV attachment to the IVC is oversewn and a new anastomosis is fashioned inferiorly [9]. The addition of a great saphenous venous cuff may be utilised in the presence of inadequate LRV length to ensure a tension-free transposition [9].

A recent study suggested 59 out of 61 patients treated by endovascular stenting had resolution of symptoms [4]. Oversized, self-expanding stents carry the least migration risk [9]. Regardless, migration, restenosis, thrombosis, and pulmonary embolism have all been reported [7, 9]. No long-term data regarding such complications in a usually young population exist [9]. Stent insertion must be followed by prolonged anticoagulation and/or antiplatelet therapy [4] which in themselves carry risk.
